# Acknowledgement of manuscript reviewers 2015

**DOI:** 10.1186/s12971-016-0071-1

**Published:** 2016-02-25

**Authors:** James Elliott Scott, Constantine Vardavas

**Affiliations:** University of Manitoba, Winnipeg, Canada; University of Crete, Gallos, Greece

## Abstract

The editors of *Tobacco Induced Diseases* would like to thank all our reviewers who have contributed to the journal in Volume 13 (2015).

**Yehia Abed**

Palestinian Territory

**Amitesh Aggarwal**

India

**Mir Faeq Ali Quadri**

Saudi Arabia

**Ti Andreeva**

Ukraine

**Sabina Antoniu**

Romania

**Stephen Apanga**

Ghana

**Thomas Arcury**

USA

**Thuy Au**

Australia

**Sunday Azagba**

Canada

**Nicola Basso**

Italy

**David Bernhard**

Austria

**Mehmet Burcu**

USA

**Andrew Busch**

USA

**Chris Butt**

USA

**Michael Byron**

USA

**P Carreiro-Martins**

Portugal

**Ping Chen**

China

**Parimal Chowdhury**

USA

**Fabio Cibella**

Italy

**Amy Cohn**

USA

**Cornelius Debpuur**

Ghana

**Kevin Delucchi**

USA

**Paul Dennis**

USA

**Joseph Difranza**

Netherlands

**Petros Dinas**

Greece

**Omara Dogar**

UK

**Pete Driezen**

Canada

**Sonia Duffy**

USA

**Frida Eek**

Sweden

**Stamatis Efstathiou**

Greece

**Konstantinos Farsalinos**

Greece

**Filippos Filippidis**

UK

**Miriam Forbes**

Australia

**Emmanuel Fort**

France

**Maria Rosaria Galanti**

Sweden

**Ferruccio Galletti**

Italy

**Ivan Gee**

UK

**Audra Gollenberg**

USA

**Semira Gonseth**

USA

**Jose Ignacio De Granda-Orive**

Spain

**Fadi Hammal**

Canada

**Reiner Hanewinkel**

Germany

**Takashi Hanioka**

Japan

**Martin Härter**

Germany

**Zhi-Hui He**

China

**Bryan Heckman**

USA

**Marita Hefler**

Australia

**Asgeir R Helgason**

Sweden

**Harri Hemila**

Finland

**Haziel Jenifer**

India

**Xavier Joya**

Spain

**Zubair Kabir**

Ireland

**M A Kaiser**

USA

**Biraj Karmacharya**

Nepal

**Akatsuki Kokaze**

Japan

**Grace Kong**

USA

**John Kraemer**

USA

**Elisabeth Kvaavik**

Norway

**Tai-Hing Lam**

Hong Kong

**Anthony Laverty**

UK

**Lambros Lazuras**

Greece

**Kwan Lee**

Korea, Republic of

**Joseph Lee**

USA

**Keir Lewis**

UK

**Zhijun Li**

China

**Li Liming**

China

**Maria J Lopez**

Spain

**Lucia Maria Lotrean**

Romania

**Stelios Loukides**

Greece

**Goedele Louwagie**

South Africa

**Ping Ma**

USA

**Mary Martinasek**

USA

**W G Mayhan**

USA

**Patricia Mcdaniel**

USA

**Matt Melvin**

USA

**Aliapsha Meysamie**

Iran

**Brennen Mills**

Australia

**Jordan Minov**

Macedonia

**Hanns Moshammer**

Austria

**Pooja Naik**

USA

**Rima Nakkash**

Lebanon

**Joel Nitzkin**

USA

**James Nonnemaker**

USA

**Hilal Ozcebe**

Turkey

**Lauren Pacek**

USA

**A Padron-Monedero**

USA

**Yue Pan**

USA

**Demosthenes Panagiotakos**

Greece

**Sophia Papadakis**

Canada

**Marcel Peloquin**

Canada

**B Piñeiro**

Spain

**Pallav Pokhrel**

USA

**Kinga Polanska**

Poland

**Evangelos Polychronopoulos**

Greece

**Shikha Prasad**

USA

**Elisa Puigdomènech**

Spain

**Sara Quandt**

USA

**Muhammad Aziz Rahman**

Australia

**Loukianos Rallidis**

Greece

**Naser Ramadani**

Kosovo

**Lars Ramstrom**

Sweden

**Nilminie Rarhnayke**

Sweden

**Mary Kay Rayens**

USA

**Antonio Rial**

Spain

**Kara Riehman**

USA

**Abraham Roos**

Sweden

**Hana Ross**

USA

**João Salgado**

Brazil

**Miguel Santibañez**

Spain

**Mohammad Abul Bashar Sarker**

Japan

**Kazunari Satomura**

Japan

**Marianne Schmid**

Germany

**Norman Schmidt**

USA

**Steven Schroeder**

USA

**David Scott**

USA

**Y Shi**

USA

**Michael Siegel**

USA

**A Sobczak**

Poland

**Andrzej Sobczak**

Poland

**Tomotaka Sobue**

Japan

**Giovanni Sotgiu**

Italy

**Gideon St.Helen**

USA

**Saso Stoleski**

Macedonia

**X Sureda**

Spain

**Steve Sussman**

USA

**Andy Tan**

USA

**Filippos Tarantilis**

Greece

**Johannes Thrul**

USA

**Sarah Thurgood**

UK

**R V Tiwari**

India

**Antigona Trofor**

Romania

**Patricia Valverde**

USA

**Antonius Van Rooij**

Belgium

**Mark Vander Weg**

USA

**Gabriela Vazquez Benitez**

USA

**J White**

New Zealand

**L Wu**

China

**Shao-Hua Xie**

Hong Kong

**He Yao**

China

**Mahmut Yardim**

Turkey

**Tong Yin**

China

**Matilde Zaballos**

Spain

**Joseph Zakhar**

USA

**Guangbiao Zhou**

China

